# Prevalence and associated factors of psychosocial distress among seafarers during COVID-19 pandemic

**DOI:** 10.1186/s12888-021-03197-z

**Published:** 2021-04-30

**Authors:** Fereshteh Baygi, Nami Mohammadian Khonsari, Arash Agoushi, Saeed Hassani Gelsefid, Armita Mahdavi Gorabi, Mostafa Qorbani

**Affiliations:** 1 Center of Maritime Health and Society, Department of Public Health, University of Southern Denmark, Esbjerg, Denmark; 2 Student Research Committee, Alborz University of Medical Sciences, Karaj, Iran; 3 Social Determinants of Health Research Center, Alborz University of Medical Sciences, Karaj, Iran; 4 Non-communicable Diseases Research Center, Alborz University of Medical Sciences, Karaj, Iran; 5 Chronic Diseases Research Center, Endocrinology and Metabolism Population Sciences Institute, Tehran University of Medical Sciences, Tehran, Iran

**Keywords:** Seafarers, Anxiety, Depression, Psychosocial distress, COVID-19

## Abstract

**Background:**

In the context of growing concerns about seafarers’ mental health during the COVID-19 pandemic, this study aimed to assess the prevalence and associated factors of psychosocial distress among seafarers of ocean-going vessels during the current health emergency.

**Methods:**

This cross-sectional study was conducted among 470 multinational seafarers working on two oil tanker international shipping companies. Psychosocial distress was assessed by using Depression-Anxiety-Stress Scale (DASS-21). General Health Questionnaire-12 (GHQ-12) and Zung Self-Rating Anxiety Scale (SAS) were used to assessed genral psychiatry disorders and self-rate anxiety. Perceived health status was assessed by a single-item question. Multivariate logistic regression was used to determine the association between demographic and work-related variables with mental health outcomes.

**Results:**

Overall, 439 out of 470 invited seafarers with a mean age of 34.5 (SD: 8.05) participated in this study (participation rate: 93.4%). The prevalence (95% confidence interval) of depression, anxiety, stress, self-rated anxiety, general psychiatric disorders, and poor perceived health status was 12.3% (9.4–15.7), 11.6% (8.7–15.0), 5.9% (3.9–8.5), 2.1% (0.9–3.8), 42.6% (38.0–47.4), and 4.3% (2.6–6.6), respectively. In the multivariate model, by increasing the duration of stay (per month) on board, the odds of depression increased by 20% (OR: 1.20 (95% CI: 1.02–1.40)). Also, non-officer seafarers experienced significantly lower psychosocial distress such as anxiety and stress levels than officers.

**Conclusion:**

High prevalence of depression, anxiety, and general psychiatric disorders among seafarers during COVID-19 was observed. Our findings also highlight the factors that need to be considered to protect seafarers’ mental well-being. Further studies to evaluate the impact of COVID-19 on psychological health issues at sea are recommended.

## Background

The COVID-19 pandemic struck the shipping industry in early March 2020. In Public Health Emergency of International Concern (PHEIC), some extraordinary restrictions, including the quarantine of ships at ports and 14-day self-isolation on international ships, were imposed by shipping companies or port authorities in order to stop the rapid spread of the disease [1]. Such limitation and specific working conditions on board [2] might cause unforeseen influences on seafarers’ psychosocial distress at sea.

Several studies have examined the influences of COVID-19 on the overall and psychosocial distress of different populations (e.g., adolescent, university students) [2–4]. Even some reviews and meta-analysis have been done on COVID-19 and its consequences [5–7]. For instance, a meta-analysis study conducted among health workers - as frontline workers- revealed that COVID-19 has a substantial impact on employees’ psychosocial distress [5]. A recently published study also revealed that half of the general population rated the psychological impact of coronavirus diseases (COVID-19) as moderate or severe [8].

Although psychosocial distress at the workplace got attention from several years ago [9, 10], psychosocial distress and well-being in the maritime industry are receiving attention lately. During the current global health emergency, employees of this hard-to-reach occupation and their challenges have been overlooked. Although the International Chamber of Shipping (ICS), the International Maritime Health Association (IMHA), and some other authorized organizations have worked to manage issues related to COVID-19 at sea, no study has been conducted in order to capture the picture of their challenges during current pandemic at sea.

The maritime industry shoulders over 90% of global trade and almost 1.6 million seafarers serving onboard ships [11]. Since they have limited access to medical and psychosocial distress services, it seems that ocean shipping and its employees are more vulnerable than the other shipping sectors or even other occupations during the COVID-19 pandemic period. Despite all mentioned above, at the time of writing, just one published study with a minimal sample size on seafarers’ well-being during COVID-19 has been found, which might not capture the full picture of such a major health emergency [12]. Besides, there has been no published study about seafarers’ overall and psychosocial distress status on ocean-going vessels.

## Materials and methods

### Study design

This cross-sectional study was conducted among 470 multinational seafarers working onboard ocean-going vessels of two international oil tanker shipping companies. In early July, all seafarers who worked on ocean-going vessels with different ranks and job categories were invitated via an invitaion letter sent to the shipping companies and participants were contacted by the shipping companies. Further follow up was done through the shipping companies.

### Data collection

All employees on board 25 ocean-going vessels with different ranks and job categories have been asked to attend the current study.

Online self-administered questionnaires, including demographic and work-related characteristics such as age, marital status, job category, and working days/hours, were used. Psychiatric distress status was also assessed by using the online Depression-Anxiety-Stress Scale-21 (DASS-21) [13], General Health Questionnaire-12 (GHQ-12) [14], and Zung Self-Rating Anxiety Scale (SAS) [15]. The participants were followed 3 times regarding completion of the questionnaires (the questionnaires were sent to all participants, to all of whom, who did not answer the questionnaires, two times more the questionnairs were sent).

All communications between the investigators of the study and the shipping companies were done via email. Then, questionnaires were sent to the vessels through email by the shipping companies. The shipping companies were asked to send the questioners through email to all vessels. Then every vessel scanned the filled-out questionnaire and sent them back to the shipping companies. The principle investigator of the study was in close contact with the shipping companies in order to access to the questionnaires and facilitate the process of follow up. All participants fluent in English who could read and understand the questions, were included in the study.

DASS-21 consists of 21-items in order to measure depression, anxiety, and stress scale. The participant can choose one of the four options; these choices are then scored based on the chosen option; a score from zero to three is given to each question. The total score in each scale that is the sum of all scores obtained from questions indicates depression, anxiety, or stress levels, and higher scores indicate a more severe mental disturbance. According to this questionnaire, DASS-21 score > 14, 7, and 9 was considered as having stress, anxiety, and depression respectively [16].

GHQ-12 is a simple tool made for the identification of general psychiatric disorders. There are 12 questions about respondents “depressive, anxiety symptoms, confidence, and overall happiness,” measured on a Likert scale (0 = less than usual, 1 = no more than usual, 2 = rather more than usual, 3 = much more than usual). The 12 questions’ values summed, resulting in a value ranging from 0 (the least psychiatric disorder) to 36 (the most severe psychiatric disorder). GHQ-12 score > 18 was defined as having general psychiatric disorders [17, 18].

Zung Self-Rating Anxiety Scale (SAS) is a 20-item questionnaire. Each is scored on a scale of 1–4 (none or little of the time, some of the time, a good part of the time, and most of the time). The raw scores range from 20 to 80. The cut-off values of the Zung questionnaire are as follows. Normal range: 20–44; Mild to moderate anxiety levels: 45–59; Marked to severe anxiety levels: 60–74; Extreme anxiety levels: 75–80 [19]. Percieived health status was assessed with a single question, “How would you describe your general health status?” the response categuries were “perfect,” “good,” “moderate,” and “poor“. For statistical analysis this variable was considered as a binary variable: good (perfect and good) and poor (moderate and poor).

### Statistical analysis

Data analysis was done by SPSS (Statistical Package for the Social Sciences software, version 16). Normality of continuous variables was checked using Kolmogrov-smirnov test. Continuous variables are presented as mean ± standard deviation (SD) and categorical variables as frequencies and percentages. Independent t-tests were used to compare mean of continuous demographic and work-related variables among subject with and without psychiatric distress. Chi-square test was used to compare frequency of categorical demographic and work-related variables with psychiatric distress. Pearson correlation coefficient was used to compare the correlation between SAS, GHQ, and DASS subscale scores. Univariate and multivariate logistic regression analysis also was used to determine the association of demographic and work-related variables (as independent variables) with psychiatric distress (as dependent variables). All variables which were significant in the univariate model were included in the multivariate model. The logistic regression analysis results are presented as the odds ratio (OR) and 95% confidence interval (CI). A *P*-value less than 0.05 was considered to be statistically significant.

## Results

Overall, 439 out of 470 invited seafarers filled out the questionnaires (participation rate: 93.4%). The mean age of participants was 34.5 (SD: 8.05). Most of the seafarers were Indian (77.7%) and married (67.9%). 53.2% of seafarers were non-officers. 51.8% of our participants worked on deck, 38.2% in the engine room, and 10% worked in the galley.

**Fig. 1:**
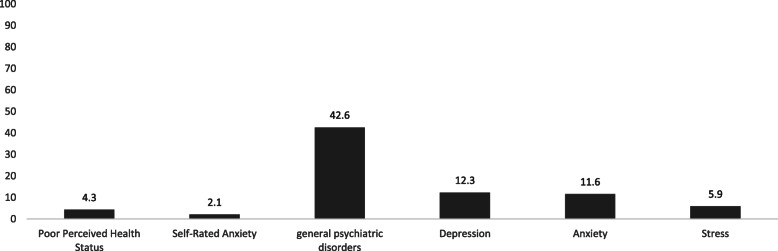
Prevalence of depression, anxiety, stress, perception of health, self-rated anxiety (SAS) and general psychiatric disorders among seafarers

Prevalence of depression, anxiety and stress was 12.3% (95% CI: 9.3–15.7), 11.6% (95% CI: 8.7–14.9) and 5.9% (95% CI: 3.9–8.5), respectively. Prevalence of self-rated anxiety was 2.1% (95% CI: 0.9–3.8), general psychiatric disorders 42.6% (95% CI 37.4–46.9) and poor perceived health status was 4.3% (95% CI: 2.6–6.6) (Fig. 1).

Married crew members had a better perception of health status compared to single ones (χ^2^: 3.85, df: 1, *p*-value: 0.05). Anxious crew members were significantly older than normal ones (t: − 2.33, df:437, *p*-value:0.02) and general psychiatric disorders were significantly greater among officers compared to non-officers (χ^2^: 22.41, df: 1, *p*-value< 0.001) (Table 1).

**Table 1:** Association of demographic and work –related variables with perceived health status, self-rated anxiety and mental disorders among seafarers

		Perceived Health Status			Self-rated anxiety			General psychiatric disorders		
		Good	Poor	***p***-value	No	Yes	***p***-value	No	Yes	***p***-value
Age (year) ^a^		34.59 ± 8.10	32.31 ± 6.61	0.22	34.36 ± 7.98	40.66 ± 9.73	0.02*	34.28 ± 8.03	35.98 ± 8.13	0.14
Work per week (hour)^a^		67.24 ± 9.64	68.47 ± 8.72	0.58	67.21 ± 9.47	71.44 ± 14.53	0.89	66.96 ± 9.21	69.61 ± 11.75	0.055
Signing in the current vessel (day)^a^		116.85 ± 63.12	115.63 ± 55.30	0.93	116.70 ± 62.82	121.55 ± 62.36	0.81	114.77 ± 62.94	119.53 ± 62.55	0.43
Marital status^b^	Single	131 (93)	10 (7)	0.05*	140 (99.3)	1 (0.7)	0.17	92 (65)	49 (35)	0.02*
	Married	289 (97)	9 (3)		290 (97.3)	8 (2.7)		160 (53.7)	138 (46.3)	
Work status^b^	Day	209 (96)	9 (4)	0.85	212 (97.2)	208 (98.6)	0.33	129 (51)	81 (49)	0.56
	Shift	203 (96)	8 (4)		6 (2.8)	3 (1.4)		119 (56.4)	92 (43.6)	
Duties^b^	Deck	213 (95)	12 (5)	0.74	218 (97)	7 (3)	0.43	126 (56)	99 (44)	0.76
	Engine	160 (96.3)	6 (3.7)		164 (99)	2 (1)		95 (57.2)	71 (42.8)	
	Service	42 (97.6)	1 (2.4)		43 (100)			27 (62.8)	16 (37.2)	
Position^b^	Officer	191 (95)	10 (5)	0.57	195 (97)	6 (3)	0.21	91 (45.3)	110 (54.7)	< 0.001*
	Other	224 (96)	9 (4)		230 (98.7)	3 (1.3)		158 (67.8)	75 (32.2)	

^a^ are reported as mean (SD), ^b^ are reported as n (%)

* statistically significant

Association of demographic and work –related variables with DASS subscales among seafarers are presented in Table 2. Average days signing in the current vessel in depressed seafarers was significantly more than other seafarers (t: − 2.42, df:411, *p*-value:0.01). Anxiety was more prevalent among officers compared to non-officers (χ^2^: 17.42, df: 1, p-value< 0.001) (Table 2). Also the prevalence of strees among officers wassignificantly higher than non-officers (χ^2^: 9.41, df: 1, p-value: 0.002).

**Table 2:** Association of demographic and work –related variables with DASS subscales among seafarers

		Depression			Anxiety			Stress		
		No	Yes	***p***-value	No	Yes	***p***-value	No	Yes	***p***-value
Age (year) ^a^		34.40 ± 8.27	35.20 ± 6.32	0.49	34.43 ± 8.1	35 ± 7.13	0.63	34.42 ± 8.12	35.65 ± 6.86	0.45
Work per week (hour)^a^		67.06 ± 9.51	68.98 ± 10.06	0.16	67.09 ± 9.50	68.86 ± 10.20	0.21	67.26 ± 9.47	67.88 ± 11.57	0.74
Signing in the current vessel (day)^a^		114.05 ± 63.68	136.25 ± 52.13	0.01*	115.19 ± 63.17	128.96 ± 58.59	0.14	115.44 ± 63.64	138.30 ± 41.68	0.07
Marital status^b^	Single	127 (90)	14 (10)	0.29	129 (91.5)	12 (8.5)	0.16	136 (96.5)	5 (3.5)	0.14
	Married	258 (86.6)	40 (13.4)		259 (87)	39 (13)		277 (93)	21 (7)	
Work status^b^	Day	188 (86.2)	30 (13.8)	0.08	194 (89)	24 (11)	0.84	203 (93.1)	15 (6.9)	0.15
	Shift	193 (91.5)	18 (8.5)		189 (89.6)	22 (10.4)		203 (96.2)	8 (3.8)	
Duties^b^	Deck	196 (87)	29 (13)	0.37	197 (87.5)	28 (12.5)	0.76	212 (94.2)	13 (5.8)	0.87
	Engine room	149 (90)	17 (10)		148 (89.1)	18 (10.9)		156 (94)	10 (6)	
	Service	36 (83.7)	7 (16.3)		38 (88.4)	5 (11.6)		40 (93)	3 (7)	
Position^b^	Officer	170 (84.6)	31 (15.4)	0.058	164 (81.6)	37 (18.4)	< 0.001*	182 (90.5)	19 (9.5)	0.002*
	Other	211 (90.5)	22 (9.5)		220 (94.4)	13 (5.6)		227 (97.4)	6 (2.6)	

^a^ are reported as mean (SD), ^b^ are reported as n (%)

* statistically significant

Correlation between SAS, GHQ and DASS scores among seafarers is presented in Table 3. The direct significant correlations was observed between all questionnaires.

**Table 3:** Pearson correlation coefficinet between SAS, GHQ-12, DASS score among seafarers

	SAS score	GHQ-12 score	Depression score	Anxiety score	Stress score
SAS score	1	0.314**	0.228**	0.230**	0.202**
GHQ-12 score		1	0.370**	0.335**	0.440**
Depression score			1	0.653**	0.782**
Anxiety score				1	0.730**
Stress score					1

*SAS* Depression-Anxiety-Stress Scale, *GHQ-12* General Health Questionnaire-12

^**^statistically significant at 0.01

Table 4 is demonstrated the association of demographic and work-related variables with psychiatric distress in logistic regression analysis among the studied population. In the multivariate (adjusted) model, by increasing per month staying on board, the odds of depression increased by 20% (OR: 1.20 (95% CI: 1.02–1.40)). Moreover, general psychiatric disorders, anxiety and stress levels were significantly lower among non- officers compared to officer (OR: 0.39 (95% CI: 0.26–0.58)), (OR: 0.21 (95% CI: 0.09–0.46)), (OR: 0.19 (95% CI: 0.06–0.60)), respectively.

**Table 4:** Association of demographic and work-related variables with psychiatric symptoms and perceived health status in logistic regression analysis among seafarers

		Perceived Health Status (poor/good)		General psychiatric disorders (yes/no)		Self-rated anxiety (yes/no)		Depression (yes/no)		Anxiety (yes/no)		Stress (yes/no)	
		Model I	Model II	Model I	Model II	Model I	Model II	Model I	Model II	Model I	Model II	Model I	Model II
Age (year)		0.96 (0.90–1.02)	(−)	1.02 (0.99–1.04)	(−)	1.08 (1.01–1.16)*	1.05 (0.96–1.16)	1.01 (0.97–1.04)	(−)	1 (0.97–1.04)	(−)	1.01 (0.97–1.06)	(−)
Work per week (hour)		1.01 (0.96–1.06)	(−)	0.98 (0.96–1)	(−)	1.04 (0.97–1.11)	(−)	1.02 (0.99–1.05)	(−)	1.01 (0.98–1.05)	(−)	1 (0.96–1.04)	(−)
Signing in the current vessel (month)		0.99 (0.79–1.23)	(−)	1.03 (0.94–1.13)	(−)	1.03 (0.75–1.42)	(−)	1.19 (1.03–1.38)*	1.20 (1.02–1.40)*	1.11 (0.96–1.28)	(−)	1.19 (1.03–1.38)*	(−)
Marital status	Single	1	(−)	1	1	1	(−)	1	(−)	1	(−)	1	(−)
	Married	0.40 (0.16–1.02)	(−)	1.61 (1.07–2.45)*	1.28 (0.74–2.21)	1.20 (0.47–3.11)	(−)	1.40 (0.73–2.68)	(−)	1.61 (0.82–3.19)	(−)	2.06 (0.76–5.58)	(−)
Work status	Day	1	(−)	1	(−)	1	(−)	1	(−)	1	(−)	1	(−)
	Shift	0.91 (0.34–2.41)	(−)	1.12 (0.76–1.64)	(−)	0.51 (0.12–2.06)	(−)	0.58 (0.31–1.08)	(−)	0.94 (0.51–1.73)	(−)	0.53 (0.22–1.28)	(−)
Duties	Deck	1	(−)	1	(−)	1	(−)	1	(−)	1	(−)	1	(−)
	Engine room	0.66 (0.24–1.81)	(−)	0.95 (0.63–1.42)	(−)	0.38 (0.07–1.85)	(−)	0.77 (0.40–1.45)	(−)	0.85 (0.45–1.60)	(−)	1.04 (0.44–2.44)	(−)
	Service	0.42 (0.05–3.33)	(−)	0.75 (0.38–1.47)	(−)	0 (0)	(−)	1.31 (0.53–3.22)	(−)	0.92 (0.33–2.54)	(−)	1.22 (0.33–4.48)	(−)
Position	Officer	1	(−)	1	1	1	(−)	1	(−)	1	1	1	1
	Non-officer	0.76 (0.30–1.92)	(−)	0.39 (0.26–0.58) *	0.36 (0.23–0.56) *	0.42 (0.10–1.71)	(−)	0.57 (0.31–1.02)	(−)	0.26 (0.13–0.50) *	0.21 (0.09–0.46) *	0.25 (0.09–0.64) *	0.19 (0.06–0.60) *

Model I: crude model, Model II: adjusted for variables which had *p*-value< 0.1 in the crude model

Values are reported as odds ratio (95% confidence interval)

^*^statisticaly significant

## Discussion

Seafarers work and live onboard for several months [20]. During the current health emergency, many seafarers trapped at sea even for more than 12 months (https://www.imo.org/en/MediaCentre/HotTopics/Pages/FAQ-on-crew-changes-and-repatriation-of-seafarers.aspx), which is against the provisions of the Maritime Labour Convention (MLC, 2006) and Standards of Training and Certification for Watchkeepers (STCW, 2010 Manila Amendment) regarding the maximum time that a seafarer can serve on board (http://www.imo.org/en/OurWork/HumanElement/,Documents/MSC.1-Circ.1598.pdf ). Due to the lack of comparative data, the current study’s findings will mostly be compared to studies done on the general population or other occupations rather than seafaring.

Our findings revealed that the prevalence of depression, anxiety, and stress was 12.3, 11.6, and 5.9%, respectively. A recently published survey (from July–September) about seafarers’ experiences during the COVID-19 pandemic revealed that more than 40% of the participants had experienced symptoms of depression and over half of the respondents reported symptoms of anxiety [21]. Such differnec between our results might be because of the period when the studies were done. Another study on Seafarers’mental health which was published in 2019, showed that 25 and 17% of seafarers completing the questionnaire had scores suggesting depression and anxiety, respectively. Authors of mentioned study believe that seafarers have higher rate of depression compare to the other occupations [22]. A study conducted on Turkish health care workers (HCW) revealed that 67.4% of physicians had depressive symptoms, 51.6% had anxiety, and almost half had stress-related symptoms [23]. Another study conducted among HCW in different Chinese hospitals showed a high prevalence of anxiety, depression symptoms, and distress [24]. Seafaring is an occupation that is isolated from society for an extended period [19]. The authors of the current study believe that this occupation’s precise nature might be one of the reasons for the observed differences in prevalence rate and associated factors of psychiatric distress. Despite such a big difference in prevalence rate, we believe that the pandemic’s psychiatric burden should not be neglected among seafarers in the long run.

In our study, the prevalence of self-rated anxiety was 2.1%, general psychiatric disorders 42.6%, and poor perceived health status was 4.3%. The high prevalence of general psychiatric disorders among seafarers was similar to studies that have been done on the general population during the COVID-19 outbreak [25, 26]. Comparative studies are needed to explore the predictors of such similarity on the prevalence of general psychiatric disorders among seafarers and the general population.

A study among adults who survived an earthquake showed that participants with lower education levels have higher GHQ-12 scores. The authors of the mentioned study believe that having a higher education level can improve knowledge about psychiatric distress disorders and might lead to taking positive action to prevent further mental disorders [27]. Our result was in contrast with the mentioned study, and officers- which have a higher level of education- had higher GHQ-12 scores compared to non-officers. We think that officers’ particular leadership role compared to non-officers in a hazardous working environment at sea might be one of the reasons for such contrast. Officers mentally are more engaged in all extraordinary situations (e.g., COVID-19) because they are the ones who should provide a safe workplace for all crew members in such dangerous situations. So, among other stressors at sea, such a deep understanding of the extent of danger for crew members should be considered a confounder.

The findings of our study will help us to protect the seafarer community as frontline workers of global trade from adverse psychiatric distress consequences of COVID-19 in the long run. Further studies to evaluate the impact of COVID-19 on psychosocial distress issues at sea are recommended. Besides, we suggest conducting qualitative research to explore predictors and patterns of underlying processes of seafarers’ psychosocial distress outcomes.

## Conclusion

Our findings show that more extended tours of duty during the COVID-19 are one of the associated factors for Psychosocial Distress (e.g., depression). Also, officers are more at risk for mental disorders, anxiety, and stress levels. To provide timely psychosocial distress well-being services for such vulnerable groups, monitoring their psychosocial distress status during different COVID-19 is highly recommended.

### Limitations and strengths of the study

The study is limited by its cross-sectional nature. Also, all limitations connected with self-report measures might affects the results of our study. It is the first study with a large sample size in maritime settings to the best of our knowledge. Also, the high response rate due to our strict follow-up was another strong point. Besides, the findings of current study will capture a full picture of the psychological problems of the target population during COVID-19 outbreak. As a result, adequate and feasible mental health services (e.g., services via the internet or hotlines) will be in place to promote seafarers’ mental health in order to achieve the United Nations Sustainable Development Goals (Goal number 3: Good Health and Well-being).

On other possible limitation is the scoring system of the GHQ-12. In different studies, the scoring has varied tremendously. This varieties can somewhat alter the result.

## Data Availability

The datasets generated and analyzed during the current study are not publicly available due to the confidential policy of the shipping companies. However, the corresponding author can be contacted if the data sets are needed for authentication or research purposes.
